# Germline *EPHB2* Receptor Variants in Familial Colorectal Cancer

**DOI:** 10.1371/journal.pone.0002885

**Published:** 2008-08-06

**Authors:** George Zogopoulos, Claus Jorgensen, Julinor Bacani, Alexandre Montpetit, Pierre Lepage, Vincent Ferretti, Lauren Chad, Subani Selvarajah, Brent Zanke, Thomas J. Hudson, Tony Pawson, Steven Gallinger

**Affiliations:** 1 Samuel Lunenfeld Research Institute, Mount Sinai Hospital, Toronto, Ontario, Canada; 2 Dr. Zane Cohen Digestive Diseases Clinical Research Centre, Mount Sinai Hospital, Toronto, Ontario, Canada; 3 McGill University and Genome Québec Innovation Centre, Montreal, Quebec, Canada; 4 Cancer Care Ontario, Toronto, Ontario, Canada; 5 The Ontario Institute for Cancer Research, Toronto, Ontario, Canada; Deutsches Krebsforschungszentrum, Germany

## Abstract

Familial clustering of colorectal cancer occurs in 15–20% of cases, however recognized cancer syndromes explain only a small fraction of this disease. Thus, the genetic basis for the majority of hereditary colorectal cancer remains unknown. *EPHB2* has recently been implicated as a candidate tumor suppressor gene in colorectal cancer. The aim of this study was to evaluate the contribution of *EPHB2* to hereditary colorectal cancer. We screened for germline *EPHB2* sequence variants in 116 population-based familial colorectal cancer cases by DNA sequencing. We then estimated the population frequencies and characterized the biological activities of the *EPHB2* variants identified. Three novel nonsynonymous missense alterations were detected. Two of these variants (A438T and G787R) result in significant residue changes, while the third leads to a conservative substitution in the carboxy-terminal SAM domain (V945I). The former two variants were found once in the 116 cases, while the V945I variant was present in 2 cases. Genotyping of additional patients with colorectal cancer and control subjects revealed that A438T and G787R represent rare *EPHB2* alleles. *In vitro* functional studies show that the G787R substitution, located in the kinase domain, causes impaired receptor kinase activity and is therefore pathogenic, whereas the A438T variant retains its receptor function and likely represents a neutral polymorphism. Tumor tissue from the G787R variant case manifested loss of heterozygosity, with loss of the wild-type allele, supporting a tumor suppressor role for *EPHB2* in rare colorectal cancer cases. Rare germline *EPHB2* variants may contribute to a small fraction of hereditary colorectal cancer.

## Introduction

Colorectal cancer is a common malignancy in Western society, with 15–20% of cases developing on the basis of apparent genetic predisposition [1]. However, the recognized familial colorectal cancer syndromes–Familial Adenomatous Polyposis (FAP), Hereditary Non-Polyposis Colorectal Cancer (HNPCC) and *MYH*-Associated Polyposis (MAP)-account for much less than one-third of inherited colorectal cancer, leaving the additional heritable component of this disease unexplained [1].

In the great majority of colorectal cancers, mutational activation of the WNT signaling pathway plays an essential role in initiating tumorigenesis [2]–[4]. Hyperactivation of WNT signaling most commonly occurs early in the adenoma to carcinoma sequence as a consequence of inactivating mutations in the *APC* tumour suppressor gene or activating oncogenic *β−catenin* mutations. These genetic alterations lead to constitutive activation of β−catenin /TCF4 complex which, in turn, drives overexpression of WNT signaling targets. These downstream effectors of the WNT pathway include genes with a critical role in the development of colorectal tumors, such as the *EPHB* and *ephrin* genes [5], [6].

A role for *EPHB2*, a member of the Eph receptor tyrosine kinase family, as a tumor suppressor gene in colorectal carcinogenesis has been suggested by several recent findings. *EPHB2* maps to a chromosomal region (1p36.1) often deleted in these tumors [7]. In addition, immunohistochemical studies have shown that expression of multiple EPHB isoforms is frequently suppressed in invasive human colorectal tumours [8]. Moreover, Alazzouzi *et al*. have recently shown a high incidence of *EPHB2* frameshift mutations in microsatellite unstable colorectal tumors and aberrant *EPHB2* promoter methylation in both microsatellite stable and unstable neoplasms [9]. Loss of EPHB2 expression in colorectal tumors has also been associated with worse prognosis [10].

Mutational inactivation of *EPHB2* has been linked to prostate carcinogenesis. Huusko *et al.* recently described a variety of *EPHB2* mutations in prostate tumours and in cell-lines [11], and a common nonsense mutation has been associated with prostate cancer in African American men with a family history of this disease [12]. These findings taken together with the established role of EPHB receptors in colorectal cancer, prompted us to hypothesize that germline *EPHB2* mutations may account for at least a fraction of the genetic alterations underlying the unexplained portion of hereditary colorectal cancer, and may represent a new cancer syndrome causing genetic predisposition to colorectal and prostate cancer. Therefore, in this study, we screened 116 population-based familial colorectal cancer cases for germline *EPHB2* mutations. The 116 probands tested had at least one additional affected first degree relative. These cases had microsatellite stable tumors, and did not meet diagnostic criteria for FAP, HNPCC and MAP. We also enriched our series with individuals who met the above inclusion criteria and had personal or family histories of prostate cancer. Our findings suggest that rare *EPHB2* alleles contribute to a small fraction of familial colorectal cancer.

## Materials and Methods

### Study Subjects and DNA Samples

Biospecimens were obtained from the Ontario Familial Colorectal Cancer Registry (OFCCR), a member of the National Cancer Institute Cooperative Family Registries for Colorectal Cancer Studies (http://epi.grants.cancer.gov/CFR/about_colon.html) [13]. The OFCCR includes 3,770 colorectal cancer cases diagnosed in the province of Ontario, Canada between 1997–2000, with an age at the time of diagnosis of 20 to 74. Age- and sex-matched control subjects with no personal history of colorectal cancer were recruited by telephone from a list of randomly selected residential telephone numbers for Ontario and from population-based Tax Assessment Rolls of the Ontario Ministry of Finance. Study subjects donated a venous blood sample and peripheral lymphocytes were isolated using Ficoll-Paque, according to the manufacturer's recommendations (Amersham Biosciences, Baie d'Urfé, Quebec, PQ, Canada). The phenol–chloroform method was used to isolate genomic DNA from lymphocytes and colorectal cancer cell-lines. The QIAamp protocol (Qiagen Inc., Mississauga, Ontario, Canada) was employed to extract genomic DNA from paraffin-embedded tissues. All study subjects signed written consent to participate in a Mount Sinai Hospital Research Ethics Board approved research study.

### *EPHB2* Mutation Screening

Using automated sequencing (Applied Biosystems 3730xl DNA Analyzer, Foster City, CA, USA), we screened for germline *EPHB2* (Entrez Gene ID: 2048; RefSeq: NM_004442, NM_017449) sequence variants in 116 familial colorectal cases (average age at diagnosis 54 years, range 22 to 74 years, 59 females, 57 males). Our analysis included patients with personal (n = 6) and/or family histories (father, n = 19; sibling, n = 23; half-sibling, n = 4) of prostate cancer. Patients with FAP, HNPCC or MAP were excluded. A series of colorectal cancer cell lines (Caco2, Colo320DM, Colo320HSR, HT29, LS513, LS1034, SW837, SW948, SW1417, T84) were also screened. We sequenced the entire coding region and at least 50 bp of intronic sequence at the exon/intron boundaries. PCR primer sequences and conditions are provided in Table S1. Variants identified are numbered relative to RefSeq NM_004442.

### A438T, D679N and G787R Allele Frequencies

A random sample of cases and matched controls from the OFCCR series were selected for studies to evaluate the A438T, D679N and G787R allele frequencies. Lymphocyte DNA samples from an additional series of OFCCR cases (n = 364 for A438T; n = 1160 for D679N; n = 182 for G787R) and population-matched controls (n = 384 for A438T; n = 1133 for D679N; n = 199 for G787R) were tested to evaluate the population frequencies of the A438T, D679N and G787R variants. Genotyping assays for the A438T, D679N and G787R variants were developed using Fluorescence Polarization-Single Base Extension (FP-SBE) [14], SNPstream [14] and RFLP, respectively (Table S2).

### Pedigree and Loss of Heterozygosity (LOH) Analyses

Testing for segregation in the families of the probands carrying the A438T and G787R variants was performed by direct sequencing, using either lymphocyte or archival DNA from paraffin-embedded tissue blocks. Loss of heterozygosity was performed by sequencing paired tumor/normal archival DNA samples and comparing sequence autoradiograms for a decrease in the intensity of the non-mutated signal compared to the mutated sequence (Table S2). These reactions were performed using the Thermo Sequenase Radiolabeled Terminator Cycle Sequencing kit, according to the manufacturer's protocol (USB Corporation, Cleveland, Ohio, USA).

### Biochemical Characterization of the A438T & G787R Variants

The *A438T* and *G787R* cDNA sequence variants were generated using PCR-based site-directed mutagenesis and RefSeq NM_004442 (OriGene Technologies Inc., Rockville, MD, USA) as the template. The PCR products were cloned into pcDNA3 (Invitrogen Canada Inc.), and sequence verified. DU145 (a gift from Dr. Irene Andrulis, Samuel Lunenfeld Research Institute) were grown in MEM/10% FBS. Cells were transiently transfected with the various *EPHB2* cDNA constructs (wild-type, *A438T*, or *G787R*) using lipofectamine 2000 (Invitrogen Canada Inc., Burlington, Ontario, Canada), according to manufacturers instructions. Five hours following transfection, medium was changed to starvation medium (MEM/0.5%FBS) for 16h. Cells were then stimulated with 2 µg/ml preclustered ephrin b1-Fc (ephrin b1-Fc, R&D Systems, Minneapolis, MN, USA; anti-Fc antibody, Jackson ImmunoResearch Laboratories Inc., West Grove, PA, USA) for 30min. Transfected EPHB2 was immunoprecipitated using anti-EPHB2 antiserum [15] and immunoblotted with anti-phosphotyrosine (4G10, Upstate Biotechnology, Lake Placid, New York, USA) or anti-EPHB2. Expression and immunoprecipitation of EPHB2 variants for *in vitro* kinase assays was performed as above except cells were left unstimulated prior to cell lysis and immunoprecipitation, as previously described by Holland *et al*. [16] Gels were analyzed and quantified using a Storm phosphoimager (Molecular Dynamics Inc., Sunnyvale, California, USA).

## Results

Mutational screening of the *EPHB2* gene in 116 patients with familial colorectal cancer identified 3 novel missense nucleotide changes and the D679N variant previously suggested by Huusko *et al.* [11] to be pathogenic in prostate cancer (Table 1). Three unrelated patients were found to carry the D679N allele, diagnosed with colorectal cancer at the ages of 57, 59 and 67 years, respectively. However, subsequent analysis of additional subjects suggests that D679N represents a rare neutral *EPHB2* polymorphism, since the variant allele was observed at similar frequencies in patients with colorectal cancer (11 out of 1133) and population-matched controls (11 out of 1160).

**Table 1:** Non-synonymous germline *EPHB2* missense changes identified in familial and random colorectal cancer cases.

Missense Change	Residue Substitution	Exon	Domain	Familial Colorectal Cancer Cases	Random Colorectal Cancer Cases	Control Subjects
nt. 1312 G→A	A438T	6	Extracellular Fibronectin TypeIII Domain	1/116	0/364	0/384
nt. 2035 G→A	D679N	11	Protein Kinase Domain	3/116	11/1160	11/1133
nt. 2359 G→A	G787R	13	Protein Kinase Domain	1/116	0/182	0/199
nt. 2833 G→A	*V945I	15	SAM Domain	2/116	Not determined	Not determined

* A conservative residue substitution (V945I).

We also screened for *EPHB2* mutations in 10 colorectal cancer cell lines, and Caco2 was found to express a novel variant, R4Q (nt. 11 A→G), in addition to the wild-type EPHB2 receptor. The R4Q amino acid substitution may cause an alteration in the signal peptide, which may lead to decreased EPHB2 expression. However, immunoprecipitation with anti-EPHB2 antiserum, followed by immunoblotting using an antiphosphotyrosine antibody, demonstrated that despite carrying the R4Q variant, the CAC02 cell line produces a functional EPHB2 receptor (data not shown).

Two of the three novel nonsynonymous variants (A438T & G787R) we identified result in biochemically significant and potentially pathogenic residue changes. The residue affected by the A438T substitution is located in the extracellular fibronectin type III domain [17]. Since this domain may be involved in ligand binding, we postulated that the A438T variant might have decreased binding activity and signaling function. The G787R variant affects a residue in the kinase domain, and this substitution might therefore directly affect the receptor's kinase and biological activity [18]. The third novel variant (V945I) was detected in two patients, and leads to a conservative substitution at the extreme carboxy-terminus in the SAM domain [19]; we have not characterized this allele further.

**Figure 1:**
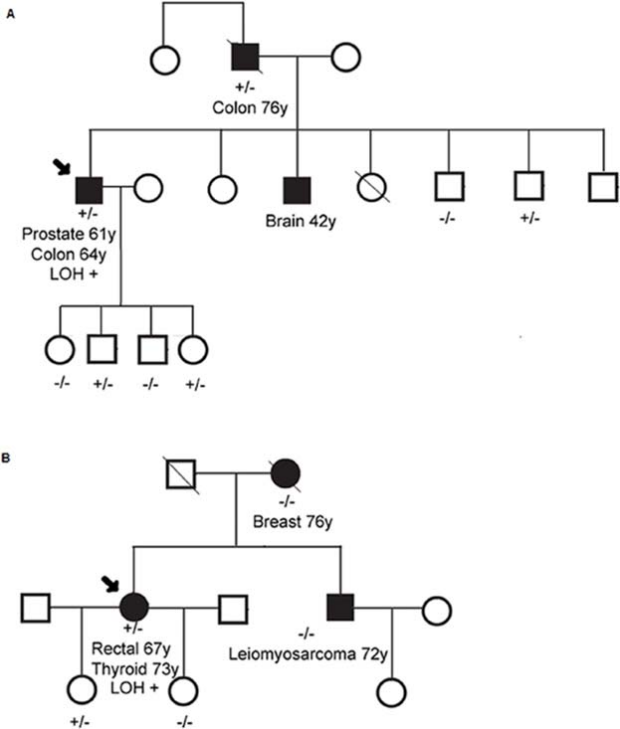
Pedigrees of colorectal cancer cases carrying the A438T (Family 1, Panel A) and G787R (Family 1, Panel B) variants. +/−, carrier; −/−, non-carrier; LOH+, Colorectal tumour tissue was found to manifest LOH, with loss of the wild-type allele; Ca, cancer.

The A438T variant was found in a patient who had two primary cancers. He was diagnosed with prostate cancer and a microsatellite stable right-sided colon cancer at the ages of 61 and 64 years, respectively (Figure 1A, Family 1). The proband's father also carried the A438T variant and is the only other family member with colon cancer, he was diagnosed with a microsatellite stable sigmoid cancer at the age of 76 years. Of the 6 unaffected family members tested for this missense change, 3 were found to carry the A438T variant (Figure 1A). Sequencing analyses of paired tumor-normal genomic DNA samples revealed loss of the wild-type *EPHB2* allele in the colon cancer from the proband, but not in the colon cancer from his father (data not shown). The father's tumor manifested LOH of the variant, rather than the wild-type, *EPHB2* allele. This latter observation may reflect the frequent loss of the 1p36.1 chromosomal region during tumorigenesis [7], [8], and not the targeted inactivation of the *EPHB2* locus by LOH.

**Figure 2:**
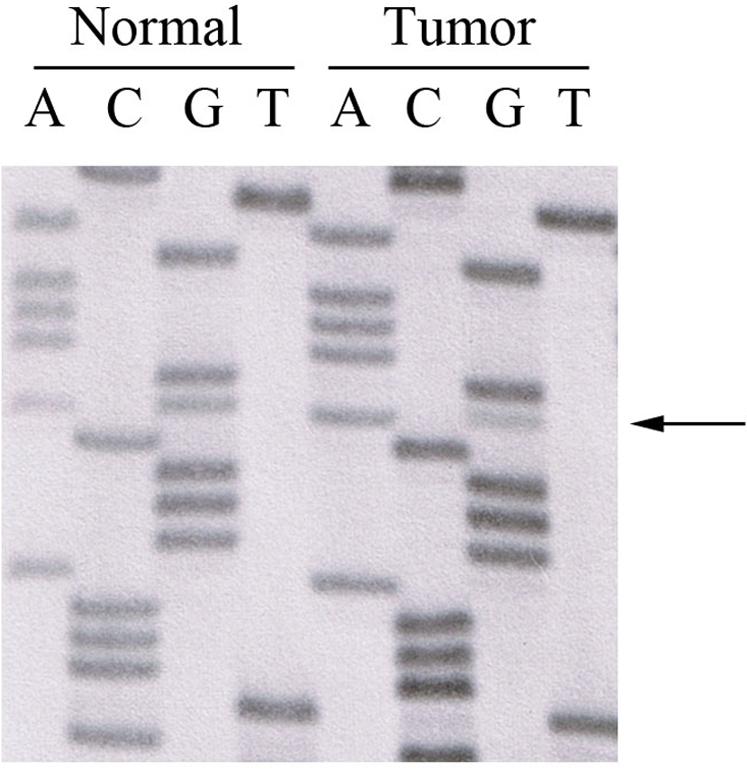
Sequencing results demonstrating loss of the wild-type *EPHB2* allele in the G787R carrier. *EPHB2* gene sequencing results of genomic DNA extracted from paired tumor and adjacent normal colon mucosa are shown. Relative to the intensities of the guanine bands in the sequencing reaction, the intensity of the guanine nucleotide at position 2359 is substantially reduced in the tumor sample, suggesting loss of the wild type allele (G) in the tumor but not in the adjacent normal tissue (arrow shows nt. 2359).

The G787R variant was detected in a patient with a diagnosis of rectal cancer at the age of 67 years (Figure 1B, Family 2). This tumor showed loss of the wild-type allele (Figure 2). The patient also reported a history of follicular type thyroid cancer at age 73 years. This proband was selected for mutation screening because of a family history of colorectal cancer on the maternal side. However, genotyping data revealed that the G787R variant allele either originates on the paternal side or is the result of a *de novo* mutation (Figure 1B). The proband's daughter is the only other carrier of this variant, and she is currently unaffected at the age of 45 years.

We estimated the allele frequencies of the A438T and G787R variants by screening an additional series of population-based colorectal cancer cases and age and sex matched control subjects. The genotyping results suggest that these two alleles are rare variants (Table 1). Neither variant was detected in control subjects and they were not identified in any additional colorectal cancer cases. Therefore, to further evaluate the possible pathogenic role the A438T and G787R receptor variants, we characterized their intrinsic tyrosine kinase activity.

**Figure 3:**
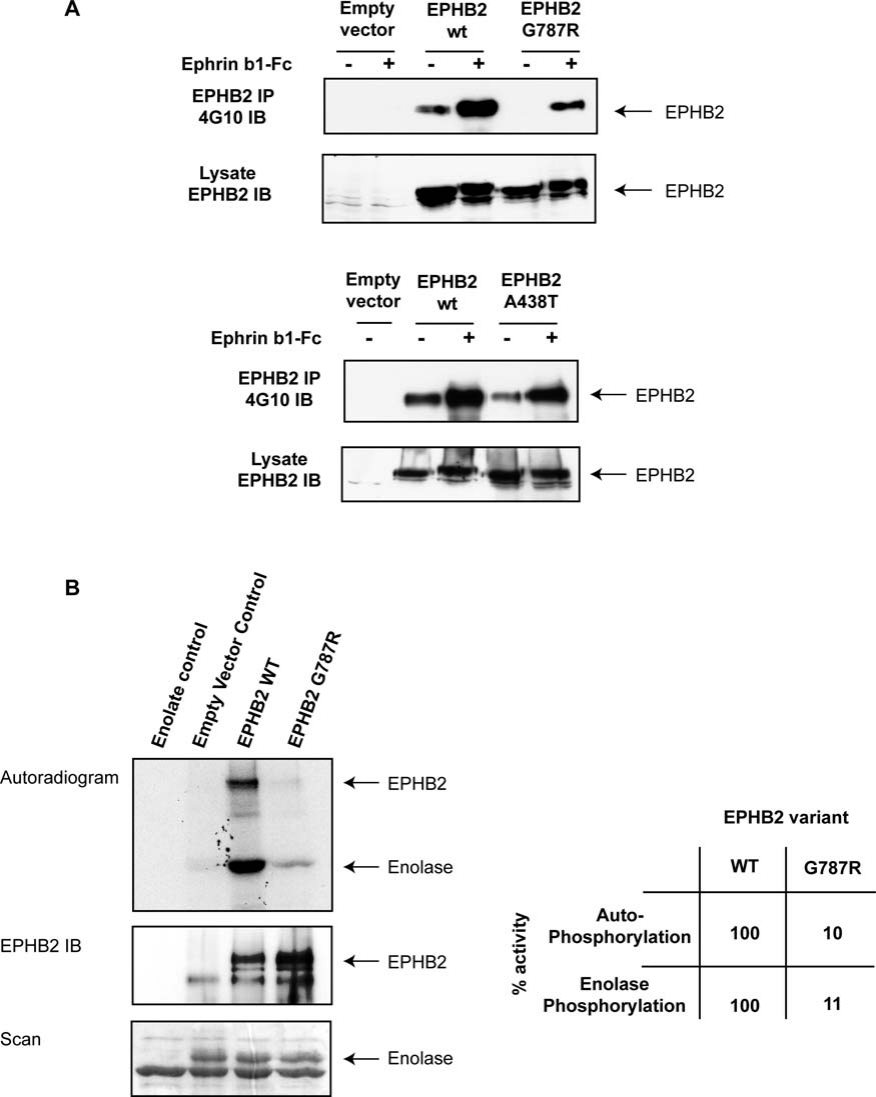
Biochemical characterization of EPHB2 variants. Panel A: Diminished autophosphorylation of EPHB2 G787R variant in response to ephrinB1 stimulation. DU145 cells were transiently transfected with cDNA constructs (empty vector; wild-type, wt; A438T; G787R) and either left unstimulated (−) or stimulated (+) with preclustered ephrinB1-Fc for 30 min. EPHB2 was immunoprecipitated (IP) and immunoblotted (IB) with antiphosphotyrosine (4G10) to evaluate receptor autophosphorylation. The cell lysate was immunoblotted with antiEPHB2 to ascertain that there was equal transfection efficiency. Panel B: Abolished kinase activity of EPHB2 G787R variant. In vitro kinase assays were performed using wild-type EPHB2 or G787R immunoprecipitates and enolase as the exogenous substrate. Prior to imaging or immonoblotting against EPHB2, phosphorylated proteins were separated by gel electrophoresis and stained with coomassie. Autoradiogram showing ^32^PγATP incorporation in EPHB2 and enolase (upper panel), anti-EPHB2 immunoblot (middle panel) and equal loading of enolase is shown (lower panel). The table shows the relative kinase activity of the wild-type EPHB2 receptor (set to 100%) *vs.* the G787R variant.

Biochemical characterization of the A438T and G787R isoforms revealed that that the G787R variant is functionally impaired, whereas the A438T change likely represents a neutral polymorphism. DU145 cells, which do not express an endogenous functional EPHB2 receptor [11], were transiently transfected with either the wild-type EPHB2 receptor or one of the two variants. We found diminished autophosphorylation of the G787R receptor, but not the A438T variant, following ephrinB1 stimulation compared to the wild-type receptor (Figure 3A). We confirmed that the G787R receptor has reduced catalytic activity by using an in vitro kinase assay. The ability of the G787R receptor to autophosphorylate or to phosphorylate the enolase substrate was approximately 9-fold lower than that of the wild-type receptor (Figure 3B), demonstrating that the G787R mutation alters receptor activity and is not a rare neutral polymorphism.

## Discussion

The Eph receptor family is the largest known subgroup of receptor tyrosine kinases. This family is further subdivided into two distinct classes, EphA (A1 to A10) and EphB (B1 to B6), based on their binding affinities for two membrane-anchored ligand families with the corresponding names of type A (A1 to A5) and B (B1 to B3) ephrins [19], [20]. Following ligand binding, Eph receptors activate cell repulsion pathways to modulate cell compartmentalization and ordered cell migration in a variety of biological processes [19].

Mouse animal model studies have shown that, in the small intestine, EphB receptors mediate intestinal stem cell proliferation [21] as well as epithelial cell migration and organization along the crypt-villous axis [5]. Since loss of mitotic activity control, epithelial patterning and tissue architecture are hallmarks of tumorigenesis, disruption of normal EPHB receptor expression and function likely promotes colorectal carcinogenesis. Constitutive EPHB receptor expression may stimulate tumor initiation by disturbing proliferative stem cell homeostasis, and secondary silencing of EPHB receptor activity may permit expansion of cancer cells, beyond the spatial boundaries imposed by intact EPHB receptor function to populate adjacent tissue structures [5], [6], [21]. In support of this hypothesis, we and others have recently shown a causal role for EphB inactivation in tumor progression [22]. We found that *EphB2* or *EphB3* silencing in Apc^Min/+^ mice results in accelerated and more aggressive colorectal tumorigenesis.

In the current study, we screened 116 population-based familial cases of colorectal cancer for mutations in a candidate tumor suppressor gene, EPHB2, and identified three candidate variants (A438T, D679N, G787R), which were further characterized. Even though the A438T allele was not observed in control subjects and was found to segregate with disease in Family 1, biochemical characterization suggests that A438T is a rare neutral polymorphism; we cannot exclude the possibility that it affects more subtle aspects of EPHB2 signaling, such as the formation of higher order oligomers. The D679N variant has been previously reported to be associated with prostate cancer [11]. Although, it remains possible that this variant modulates predisposition to prostate cancer, our data suggest that it occurs at a population frequency of approximately 1% and that it does not, on its own, increase susceptibility to colorectal cancer. We observed the D679N allele in a similar number of patients with colorectal cancer and population-matched controls. In contrast to these latter variants, our data suggest that the G787R variant is functionally compromised and may be a rare cause of hereditary colorectal cancer. The G787R variant was identified in a patient diagnosed with rectal cancer at 67 years of age, and biochemical characterization revealed that the G787R substitution markedly diminishes the receptor's intrinsic kinase activity.

There have been two other investigations examining the contribution of germline *EPHB2* mutations to colorectal cancer susceptibility. Oba *et al.* screened for *EPHB2* mutations in colon tumors and respective normal colon tissues from 50 patients with colorectal cancer, and identified an intron 8 alteration in a single tumor sample, which results in a nonsense mutation. However, it is unclear if this is a somatic mutation, as there is no indication whether this genetic change was also observed in the paired normal colon sample [23]. This investigation also identified 15 cases with LOH involving the *EPHB2* gene and screened for mutations in the remaining *EPHB2* allele. Since mutations in the remaining allele were not identified, Oba *et al.* suggested that *EPHB2* is not a classical tumor suppressor gene. However, since only 50 samples of likely sporadic cases of colorectal cancer were analyzed, a tumor suppressor role for *EPHB2* cannot be excluded. In a more recent study, Kokko *et al.* reported an association of three novel variants with colorectal cancer [24]. Germline missense changes resulting in I361V, R568W, and D861N were observed in colorectal cancer patients, but not in healthy controls. However, it is possible that these three variants are rare neutral polymorphisms since the biological significance of the variants was not evaluated using direct functional assays. The patients screened in these latter two studies did not necessarily have significant family histories of colorectal cancer. In contrast to these two earlier reports, our study was designed to specifically evaluate the role of germline *EPHB2* mutations in patients with familial colorectal cancer, and not in sporadic cases. Despite study design differences, together these three investigations suggest that *EPHB2* germline mutations are not common occurrences in colorectal cancer. Further investigations of larger sample sizes are needed to confirm this observation.

In summary, we identified a germline *EPHB2* variant (G787R) with diminished biological activity in a colorectal cancer patient, and suggest that *EPHB2* mutations contribute to a small fraction of hereditary colorectal cancer. The rarity of germline *EPHB2* mutations supports a more significant role for EPHB2 in colorectal tumor progression rather than in tumor initiation. Since the EPHB receptors (EPHB2, EPHB3 and EPHB4) follow a similar pattern of transcriptional silencing in colorectal cancers, all EPHB receptor family members probably play a similar role in this disease. Therefore, the EPHB family likely accounts for a minor proportion of genetic predisposition to colorectal cancer but has an important role in tumor progression. Although our findings suggest that the *EPHB* gene family should not be routinely screened for germline mutations in familial cases, the *EPHB* genes are candidate tumor suppressors, likely accounting for rare cases of familial colorectal cancer.

## Supporting Information

Table S1 (0.06 MB DOC)

Table S2 (0.05 MB DOC)
