# Supplementary material for: Germline EPHB2 Receptor Variants in Familial Colorectal Cancer
Source: PLoS One. 2008 Aug 6;3(8):e2885. doi: 10.1371/journal.pone.0002885 (PMC2483346; doi:10.1371/journal.pone.0002885)
Supplement: Table S1 — (0.06 MB DOC) [file pone.0002885.s001.doc]

|  |  |  |  |
| --- | --- | --- | --- |
| **Table S1.** PCR primers and conditions used to generate PCR fragments for automated sequencing analyses. | | | |
|  |  |  |  |
| **Primer Name**  F= forward primer  R= reverse primer | **Sequence** | **PCR conditions:**  MgCl_2_ Concentration, annealing temperature,  El elongation time, number of cycles, additional reagents  (96^o^C x 30 sec, X^o^C x 1min, 72^o^C x XX min) | |
|  |  |  |  |
| EPHB21F | gcttcaagagacgacccaac | 1.5mM Mg, 60**^o^**C, 90 sec, 35 cycles | |
| EPHB21R | gagcccctcactgcagact |
|  | | | |
| EPHB22F | agacaggtccattccacagc | 1.5mM Mg, 60**^o^**C , 90 sec, 35 cycles | |
| EPHB22R | cgcagcagtggtctctcc |
|  | | | |
| EPHB2exon01Fnv | ccctgccctgacaggtagt | 1.5mM Mg, 58**^o^**C, 120 sec, 37 cycles  (10% DMSO;1 M betaine) | |
| EPHB23R | cgctcgctcacCTTCCAC |
|  | | | |
| EPHB2exon01F | ctggatggctcattctgctg | 1.5mM Mg, 60 **^o^**C, 90 sec; 40 cycles  (10% glycerol; 5%DMSO) | |
| EPHB2exon1Rnv | ctacaaagcctcggcaagat |
|  | | | |
| EPHB2exon02F | gagggcccaacagaaagatt | 1.5mM Mg, 60 **^o^**C, 90 sec, 35 cycles | |
| EPHB2exon02R | tccagcctgttgtcatggta |
|  | | | |
| EPHB2exon03F | ccaccttagactgagtgtgtgc | 1.5mM Mg, 58 **^o^**C, 90 sec, 35 cycles | |
| EPHB2exon03R | agtgcctcatggtcagctct |
|  | | | |
| EPHB2exon04F | cctggagacttcaaacccttc | 1.5mM Mg, 60 **^o^**C , 90 sec, 35 cycles | |
| EPHB2exon04R | ccagggaaagactggctgta |
|  | | | |
| EPHB2exon05F | gtggggagacaggacaaaga | 1.5mM Mg, 60 **^o^**C , 90 sec, 35 cycles | |
| EPHB2exon05R | aacaaatgggagcaattgttatt |
|  | | | |
| EPHB2exon06F | acccatagccctgcatga | 1.5mM Mg, 60 **^o^**C , 90 sec, 35 cycles | |
| EPHB2exon06R | cctgcctgaggtctcacagt |
|  | | | |
| EPHB2exon07F | tttatccaatggccagacct | 1.5mM Mg, 60 **^o^**C , 90 sec, 35 cycles | |
| EPHB2exon07R | acaacgggaacagaaacagg |
|  | | | |
| EPHB2exon08F | gggcatgtaacaggtgctct | 3.0mM Mg, 64 **^o^**C, 90 sec, 35 cycles | |
| EPHB2exon08R | tatcccaaagggatggattg |
|  | | | |
| EPHB2exon09F | gacttgaggtgggaggagtg | 1.5mM Mg, 60 **^o^**C , 90 sec, 35 cycles | |
| EPHB2exon09R | cttgcaaaggcctagaggtg |
|  | | | |
| EPHB2exon10F | ctggtctctgttcccctcac | 1.5mM Mg, 60 **^o^**C , 90 sec, 35 cycles | |
| EPHB2exon10R | tcccacatcctaccttctgc |
|  | | | |
| EPHB2exon11F | cagccagggataaccagaga | 1.5mM Mg, 60 **^o^**C , 90 sec, 35 cycles | |
| EPHB2exon11R | agcttttccaagaccccttc |
|  | | | |
| EPHB2exon12F | cctgctctggtttcccatta | 1.5mM Mg, 60 **^o^**C , 90 sec, 35 cycles | |
| EPHB2exon12R | aattgggcgttagtgaaagtg |
|  | | | |
| EPHB2exon13F | cacagggtgggaggattaag | 1.5mM Mg, 58 **^o^**C , 90 sec, 35 cycles | |
| EPHB2exon13R | atgggtggcctttcctactt |
|  | | | |
| EPHB2exon14F | tggggagaagctatctcagg | 1.5mM Mg, 60 **^o^**C , 90 sec, 35 cycles | |
| EPHB2exon14R | cggccttcctttggtaattt |
|  | | | |
| EPHB2exon15F | tggggagaagctatctcagg | 1.5mM Mg, 60 **^o^**C , 90 sec, 35 cycles | |
| EPHB2exon15R | cggccttcctttggtaattt |
|  | | | |
| EPHB2exon16-17F | gctcttccacctcacaccat | 1.5mM Mg, 60 **^o^**C , 90 sec, 35 cycles | |
| EPHB2exon16-17R | CCTGTCTCTCCCCTTCACAT |
|  | | | |
| EPHB2exon17bF | AGATTCATGCGATGTGTCCA | 1.5mM Mg, 60 **^o^**C , 90 sec, 35 cycles | |
| EPHB2exon17bR | GCCCTTGGTGTATTGCCTAA |
|  | | | |
| EPHB2exon17cF | GCTGAGAAGGGTTGATCCTG | 1.5mM Mg, 60 **^o^**C , 90 sec, 35 cycles | |
| EPHB2exon17cR | TAGGCTCCCAGAGGGAAAAC |
