# Supplementary material for: Germline EPHB2 Receptor Variants in Familial Colorectal Cancer
Source: PLoS One. 2008 Aug 6;3(8):e2885. doi: 10.1371/journal.pone.0002885 (PMC2483346; doi:10.1371/journal.pone.0002885)
Supplement: Table S2 — (0.05 MB DOC) [file pone.0002885.s002.doc]

| **Table S2. Primer and Reaction Conditions for Genotyping and Radiolabled Sequencing Assays** | | | |
| --- | --- | --- | --- |
|  |  |  |  |
|  |  |  |  |
| **Missense** | **Primer Name** | **Sequence** | **Reaction Conditions:** |
| **Genotyped/Sequence** | (F= forward, R= reverse) |  |  |
|  |  |  |  |
| **nt. 1312 G-->A (A438T)** | EPHB2exon06F2 | CCACCTGTCCGAGTACCTCT | 2.5 mM MgCl2, 94^o^C 40 sec, 58^o^C 40 sec, 72^o^C 40 sec,  35 cycles |
| Thermo Sequenase Radiolabeled Terminator Cycle Seqiuencing | EPHB2exon06R2 | TAGTCCAGGATCACGCCATT |  |
|  |  |  |  |
| **nt. 1312 G-->A (A438T)** | EPHB2exon06F | ACCCATAGCCCTGCATGA | 3.0 mM MgCl2, 95^o^C 30 sec, 59^o^C 30 sec, 72^o^C 30 sec,  45 cycles |
| FP-SPE Genotyping | EPHB2exon06R3 | CCTGAGGTCTCACAGT | PCR products were cleaned using Exonuclease I, Shrimp alkaline phosphatase and pyrophosphatase at 37 ^o^C for 45 min, then inactivated at 95^o^C for 25 min. |
|  | EPHB2exon06A438T (Probe) | GCCTCTCTTCCAGCTCCATCG | SBE reaction performed using 0.25uM probe, acycloterminator nucleotides and acyyclopolymerase at 54^o^C for 45 cycles. |
|  |  |  |  |
|  |  |  |  |
| **nt. 2035 G-->A (D679N)** | EPHB2exon11F2 | TGGCCATCAAGACGCTCAAGTC | 3.0 mM MgCl2, 95^o^C 30 sec, 55^o^C 55 sec, 72^o^C 30 sec,  40 cycles |
| SNPstream Genotyping | EPHB2exon11R2 | AGGGAGCCATTCTCCATGAACTCG | PCR products were treated with exonuclease I, shrimp alkaline phosphatase at 37^o^C for 30 min, then inactivated at 95^o^C for 10 min. Extension reactions and hybridizations to the Orchid UHT microarray plates were then performed. |
|  | EPHB2exon11F-U9 | GACCTGGGTGTCGATACCTACGAAG  CCTCCATCATGGGCCAGTTC |  |
|  |  |  |  |
|  |  |  |  |
| **nt. 2359 G-->A (G787R)** | EPHB2exon13F2 | CACAGGGTGGGAGGATTAAG | 2.5 mM MgCl2, 94^o^C 40 sec, 58^o^C 40 sec, 72^o^C 40 sec,  35 cycles |
| RLFP Genotyping | EPHB2exon13R2 | TCCGGTACTGGATGGCTTC | nt. 2359 G-->A abolishes EciI restriction site in PCR product |
| Thermo Sequenase Radiolabeled Terminator Cycle Seqiuencing |  |  |  |

|  |  |  |  |
| --- | --- | --- | --- |
|  |  |  |  |
|  |  |  |  |
|  |  |  |  |
|  |  |  |  |
|  |  |  |  |
|  |  |  |  |
|  |  |  |  |
|  |  |  |  |
|  |  |  |  |
|  |  |  |  |
